# Perceptions and Interest in Healthy Longevity Medicine Among Adults in Nigeria: A Cross‐Sectional Online Survey

**DOI:** 10.1002/puh2.70443

**Published:** 2026-11-13

**Authors:** Shuaibu Saidu Musa, Mohamed Mustaf Ahmed, Bukola Lawal, Angella Maliakini Katenga, Krit Pongpirul

**Affiliations:** ^1^ Center of Excellence in Preventive and Integrative Medicine (CE‐PIM) Faculty of Medicine Chulalongkorn University Bangkok Thailand; ^2^ Graduate Program in Clinical Sciences Faculty of Medicine Chulalongkorn University Bangkok Thailand; ^3^ Department of Nursing Science Ahmadu Bello University Zaria Nigeria; ^4^ Faculty of Medicine and Health Sciences SIMAD University Mogadishu Somalia; ^5^ Graduate Program in Medical Sciences Faculty of Medicine Chulalongkorn University Bangkok Thailand; ^6^ School of Global Health and Department of Preventive and Social Medicine Faculty of Medicine Chulalongkorn University Bangkok Thailand; ^7^ Clinical Research Center Bumrungrad International Hospital Bangkok Thailand; ^8^ Department of Infection Biology & Microbiomes, Faculty of Health and Life Sciences University of Liverpool Liverpool UK

**Keywords:** aging, healthspan, healthy longevity medicine, lifespan, Nigeria, perception, religion

## Abstract

**Background:**

Population aging in Nigeria underscores the need for healthy longevity medicine (HLM), yet little is known about how religious affiliation is associated with perceptions of and interest in HLM services.

**Objective:**

To assess the association of religion and age with Nigerians' perceptions of and interest in HLM.

**Methods:**

An online cross‐sectional survey was conducted among Nigerian adults using convenience and snowball sampling through social media. Of 30,408 survey submissions, 29,996 were excluded as likely bot entries because they included only a timestamp and no substantive questionnaire answers. After screening and cleaning, 400 valid responses were analyzed.

**Results:**

Among 400 respondents (mean age 30.9 ± 7.7 years), 52.8% were Muslim and 47.2% Christian. Christians had higher HLM perception mean scores than Muslims (4.39 ± 1.01 vs. 4.04 ± 1.09; Mann–Whitney *U*, *z* = 3.882; *p* < 0.001). Perception did not differ by age group (*p* = 0.188). Religious affiliation was not associated with interest in HLM services (adjusted odds ratio [aOR] 0.84, 95% confidence interval [CI] 0.41–1.73; *p* = 0.642). Ages 31–45 had higher unadjusted odds of interest (OR 2.08, 95% CI 1.06–4.07; *p* = 0.033), but not after adjustment (aOR 2.03, 95% CI 0.94–4.36; *p* = 0.070). After adjustment, undergraduates had lower odds of interest than those with a diploma (aOR 0.36, 95% CI 0.14–0.93; *p* = 0.036). Compared with income ≤₦300,000/month, earning ₦301,000–₦500,000 (aOR 0.31, 95% CI 0.11–0.90; *p* = 0.032) or not reporting income (aOR 0.30, 95% CI 0.12–0.76; *p* = 0.011) was also linked to lower interest odds.

**Conclusion:**

Religious affiliation was associated with differences in HLM perceptions but not with interest in HLM services. Education and income, rather than religion or age, were the factors most clearly associated with interest, highlighting the importance of culturally sensitive communication.

## Graphical abstract

Healthy longevity medicine (HLM) is an emerging preventive healthcare approach focused on extending the years lived in good health by delaying age‐related decline and promoting long‐term well‐being. However, public perceptions of and interest in HLM services remain poorly understood in Nigeria and other sub‐Saharan African settings. This graphical abstract presents findings from a nationwide online cross‐sectional survey of 400 Nigerian adults aged ≥18 years conducted between November and December 2025. The study examined the associations of religious affiliation and age group with perceptions of and interest in HLM, while adjusting for education, monthly income, and geographic region. Christians reported significantly higher HLM perception scores than Muslims (mean difference = 0.35; Mann–Whitney *z* = 3.882; *p* < 0.001), whereas perceptions did not significantly differ across age groups. Overall interest in HLM services was high (87.3%), but religious affiliation and age were not independently associated with interest after adjustment. Instead, educational level and monthly income were the factors associated with differences in interest in HLM services. These findings highlight a distinction between factors associated with how HLM is perceived and factors associated with willingness to engage with HLM services. Understanding these relationships may support culturally appropriate communication, equitable access strategies, and effective implementation of HLM initiatives in Nigeria and similar sub‐Saharan African contexts.

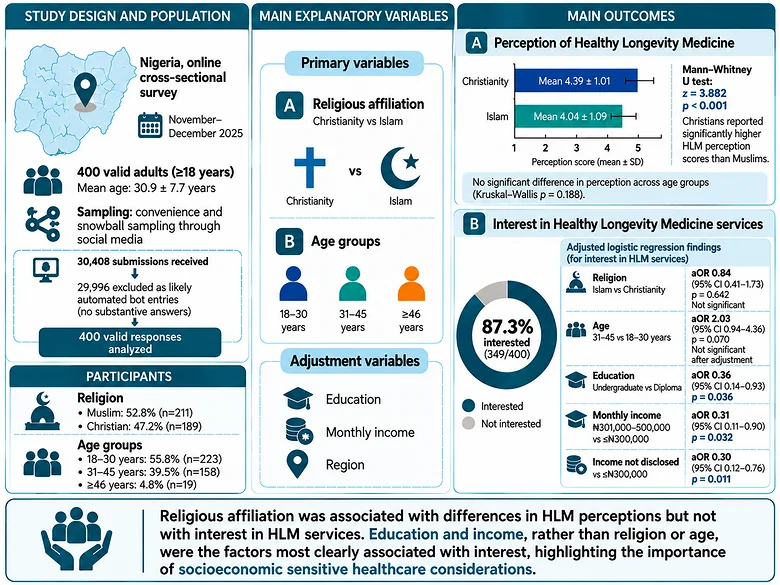

## 1 Introduction

Global population aging is accelerating, prompting renewed attention to public health strategies that address not only lifespan (the total years lived) but also healthspan, defined as the years lived in good health. The widening gap between lifespan and healthspan is a growing concern; globally, this gap is estimated to average approximately 9 years, underscoring the need for approaches that promote healthier aging rather than longevity alone [1]. This global average requires careful qualification when applied to Nigeria. Life expectancy at birth in Nigeria is roughly 54–56 years, far below the global figure. Therefore, both the number of years at stake and the ages at which ill health accumulates differ from the global perspective. Nigeria also lacks the surveillance needed to measure healthspan directly. There is no national longitudinal study of aging comparable to those in Europe or North America, and WHO records that death registration data for Nigeria are not usable on quality grounds. The reported healthy life expectancy of approximately 54.9 years [2] is therefore modeled rather than observed, and WHO cautions against using such estimates to compare countries [3]. Any national healthspan gap figure for Nigeria should therefore be read as indicative rather than measured. The size of this gap matters for healthy longevity medicine (HLM) in two ways. Where the gap is large, more years are lived with disease and disability, so the potential benefit of interventions that delay functional decline is also greater. The same setting makes implementation harder. Limited surveillance makes it difficult to identify who needs these interventions and to judge whether they work, and constraints on health system capacity and financing may limit who can receive them. The healthspan gap in Nigeria is therefore both a reason to consider HLM and a challenge to its delivery.

Despite this limited measurement, HLM, which integrates biological, clinical, and preventive strategies to delay functional decline and chronic disease, has emerged as a promising response to this challenge. Demographic projections highlight the urgency of this agenda. By 2030, one in six people worldwide is expected to be aged 60 years or older, with the global population of older adults increasing from 1.0 billion in 2020 to 1.4 billion [4]. By 2050, this number is projected to reach 2.1 billion, whereas the population aged 80 years and above may triple to more than 426 million [4, 5]. Although population aging has been most pronounced in high‐income countries such as Japan, where approximately 30% of the population is already aged 60 years or older, low‐ and middle‐income countries are experiencing the most rapid demographic shifts. These countries are projected to account for nearly two‐thirds of the global population aged 60 years and above by 2050 [4].

In Africa, where more than 60% of the population is currently under 25 years of age, population aging is an emerging but increasingly critical public health issue. Although the continent presently has the smallest proportion of older adults globally, the number of individuals aged 60 years and above is expected to triple by 2050, representing an increase of approximately 250% [6, 7]. In Nigeria, the most populous country in Africa, life expectancy has risen from approximately 47 years in 2000 to about 55 years in recent estimates [6]. However, improvements in healthspan have not kept pace with gains in lifespan, creating a growing burden of chronic disease, disability, and unmet healthcare needs among older adults [8, 9].

HLM offers a framework for addressing these challenges by emphasizing disease prevention, early intervention, and maintenance of functional capacity across the life course [10]. As interest in longevity‐focused healthcare grows, attention has increasingly turned to the social and cultural factors that influence how such approaches are perceived and adopted. Religion is one of the most influential of these factors. Religious beliefs have been shown to shape health behaviors, attitudes toward medical care, and acceptance of lifestyle modifications across diverse settings [11].

Nigeria provides a particularly important context for examining the intersection of religion and healthy aging. With a population exceeding 230 million, the country is characterized by substantial religious diversity, primarily comprising Christianity and Islam alongside indigenous belief systems [12, 13, 14]. Religion is closely bound up with how illness, treatment, and longevity are interpreted. Some literature associates Islamic thought with an emphasis on divine will in matters of life and health, which may bear on attitudes toward interventions intended to extend lifespan or alter aging trajectories [15, 16]. Other work associates Christian traditions with an emphasis on holistic well‐being, which may sit more comfortably with interventions framed around spiritual and physical flourishing [17, 18]. These are tendencies reported in particular settings rather than defining features of either faith, and the contrast should not be drawn too sharply. Both traditions hold that life is divinely given, and both have well developed accounts of bodily and spiritual flourishing. Much of this literature also comes from outside Nigeria, so how far it transfers to this setting is uncertain.

Despite increasing global interest in longevity medicine, empirical evidence on how religious affiliation relates to perceptions of and interest in HLM remains limited, particularly in low‐ and middle‐income countries. This gap complicates the development of culturally and religiously responsive policies for aging populations. Comparative perspectives from settings such as Singapore, where a parallel healthy longevity (HELO) survey has been conducted, further underscore the need for context‐specific approaches in Nigeria, because sociocultural determinants differ substantially. This study is therefore exploratory.

This study examines how religious affiliation (specifically Christianity and Islam) and age are associated with Nigerians’ perceptions of and interest in HLM. By exploring these relationships, the study aims to inform culturally sensitive public health communication and policy strategies that support the equitable integration of HLM into Nigeria's evolving health system and contribute to the broader discourse on healthy aging in diverse sociocultural settings.

## 2 Methods

### 2.1 Study Design and Setting

A nationwide online cross‐sectional convenience survey was conducted in Nigeria between November and December 2025. For descriptive purposes, the country was considered according to its two broad geographical regions (North and South).

### 2.2 Study Population and Eligibility Criteria

The study target population comprised Nigerian adults aged 18 years and older. Eligible participants were Nigerian residents who were able to read English, had internet access, and provided informed consent. Individuals residing outside Nigeria and those who did not identify as either Christian or Muslim were excluded from the study.

### 2.3 Sample Size

A target of 400 respondents was utilized as a pragmatic recruitment goal rather than the product of a formal power calculation. It reflected the number of completed responses judged achievable through voluntary sharing of the survey link, within the study period. Because participants were recruited by snowball sampling rather than by random selection from a national sampling frame, the achieved sample carries no margin of error and affords no population‐level precision.

### 2.4 Sampling Strategy and Recruitment

Participants were recruited using a nationwide snowball sampling approach. The survey link was disseminated via major social media platforms, including WhatsApp, X (formerly Twitter), LinkedIn, and Facebook. Respondents were initially targeted from the six geopolitical zones in Nigeria (six persons each), and they were encouraged to share the survey within their networks to increase reach and geographic diversity. These seed participants were selected due to their wide networks and convenience of access across religion, ethnicity, and region. No incentives were offered. We also publicized the survey on our own social media accounts, reaching networks in both Southern and Northern Nigeria and followers of both religious groups, with periodic reminders. Our accounts had over 6000 friends and followers across WhatsApp, X (formerly Twitter), LinkedIn, and Facebook. The estimated reach of these posts was not, however, recorded.

Probability sampling was not feasible with the resources available. Nigeria has no publicly accessible national sampling frame of adults with contact details, and a probability‐based household survey spanning all regions would have required field teams and funding beyond the scope of this unfunded study. Online snowball recruitment was chosen because it allowed national reach at low cost and over a short period.

### 2.5 Data Collection Tool and Survey Administration

Data were collected using an online questionnaire hosted on Google Forms. The survey was administered in English and required approximately 15–20 min to complete. The questionnaire comprised four sections: (A) sociodemographic characteristics; (B) knowledge related to lifespan and healthspan; (C) awareness and perceptions of HLM and related health priorities; and (D) daily activities, social environment, personality, experiences, and opinions.

The questionnaire was developed by the HELO consortium, a research collaboration spanning several universities and coordinated largely from the National University of Singapore. Members across the participating institutions contributed to the HELO conceptual framework, which integrates lifespan, healthspan, and HLM concepts. The questionnaire was aligned with this framework and was validated for global use. Survey items were informed by existing literature and expert input across geroscience, medicine, and public health, with attention to conceptual clarity and contextual relevance.

### 2.6 Study Measures and Variables

Religion and age group were treated as primary explanatory variables. Outcome measures included (A) interest in HLM services, analyzed as a binary (yes/no), and (B) perception scores regarding HLM, treated as a continuous scale‐derived variable for group comparisons.

The perception score was derived from Section C of the questionnaire, which covered awareness and perceptions of HLM and related health priorities. Items were rated on a 5‐point Likert scale running from “definitely not” to “definitely yes.” The score was computed as the arithmetic mean of the contributing items, rather than as a summed total or a factor score, so it retains the one to five metric of the underlying items and is directly interpretable on that scale. All contributing items were scored in the same direction before averaging. Higher scores indicate a more favorable view of HLM. A score of 4 therefore corresponds to a broadly positive orientation and a score of 2 to a broadly negative one.

The perception score comprised three items. Each item asked how willing the respondent would be to accept one of three conceptually distinct interventions: increasing lifespan alone, increasing healthspan alone, and increasing both lifespan and healthspan. Cronbach's alpha for these three items was 0.60, both unstandardized and standardized. The mean inter‐item correlation was 0.31, and item‐rest correlations ranged from 0.34 to 0.44 from the 400 responses. The Cronbach's alpha is below the conventional threshold of 0.70 and is acknowledged as a limitation of the perception score under Section 5.

### 2.7 Data Management and Statistical Analysis

Survey data were downloaded, cleaned, and analyzed using Stata version 19 SE. Descriptive statistics, including frequencies, percentages, means, and standard deviations, were used to summarize participant characteristics and survey responses. The distribution of the perception score was assessed with the Shapiro–Wilk test, which indicated a departure from normality (*W* = 0.94292, *z* = 6.554, *p* < 0.001; *n* = 400). Perception scores were therefore compared between religious groups using the Mann–Whitney *U* test and across age groups using the Kruskal–Wallis test. Means, standard deviations, and 95% confidence intervals are reported for descriptive purposes. Factors associated with interest in HLM services, the binary dependent variable, were examined using logistic regression, with religious affiliation and age group as independent variables, adjusted for education level, monthly income, and region as potential confounders. Calibration of the multivariable model was assessed with the Hosmer–Lemeshow goodness‐of‐fit test using 10 groups, and discrimination with the area under the receiver operating characteristic curve (AUC). Multicollinearity among the independent variables was assessed with variance inflation factors (VIFs), using the same reference categories and observations as the multivariable model. Willingness‐to‐pay responses for HLM services were also captured. These data were collected but were not analyzed in the present study, and no willingness‐to‐pay result is reported, which limits the study's scope on affordability.

The survey generated 30,408 submitted responses, which underwent a structured data screening and cleaning process before statistical analysis. Response records were monitored from the time the survey link was distributed to identify potential data integrity concerns. The initial screening stage focused on identifying incomplete or potentially automated submissions. Records that contained only the submission timestamp and no substantive questionnaire responses were excluded from further consideration. Following this screening, 412 responses were identified as containing substantive and complete survey information suitable for verification.

A second stage of data cleaning was conducted to ensure the reliability and completeness of the dataset. During this process, 12 responses were removed based on predefined exclusion criteria, including incomplete questionnaire entries and data quality concerns identified during verification. This resulted in a final analytical sample of 400 valid responses used for subsequent analysis. A summary of the response screening and cleaning process is presented in Figure 1.

**FIGURE 1:**
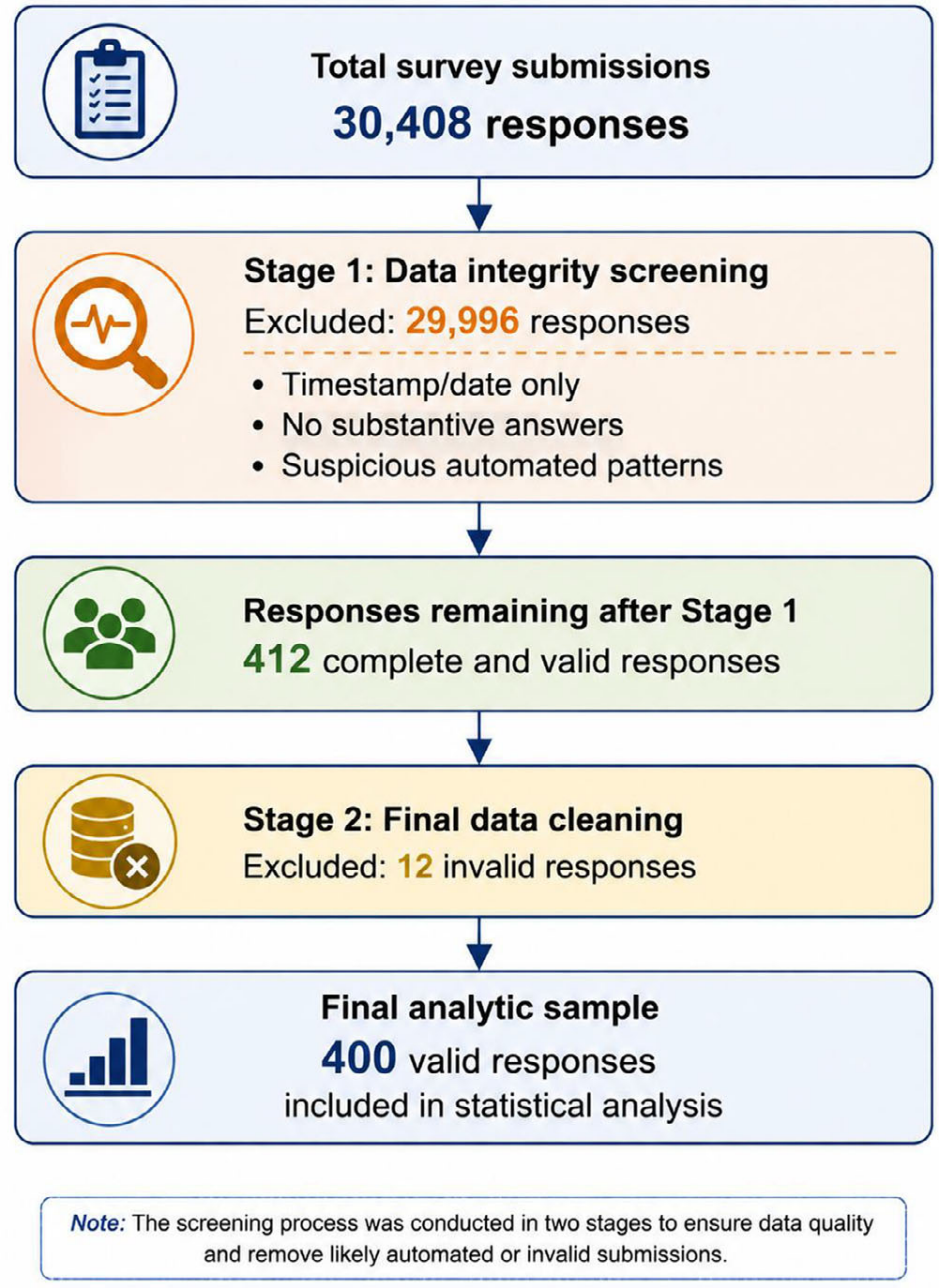
Flowchart of survey response screening and data cleaning process (*N* = 400). The flowchart illustrates the two‐stage screening procedure used to ensure data quality of the 30,408 survey submissions received.

Considerations for the high volume of submissions were considered. Viral sharing by genuine respondents would be expected to produce completed questionnaires. Accidental repeat submission by genuine respondents would be expected to produce duplicate records.

### 2.8 Ethical Considerations

The study was conducted in accordance with the Belmont Report principles of respect for persons, beneficence, and justice. Participants received an electronic information sheet and provided informed consent digitally before accessing the survey. Participation was voluntary, and respondents could withdraw at any time without consequence. No personally identifiable information was collected. The study was considered minimal risk, as it focused on public health knowledge and attitudes. Ethical approval was obtained from the Institutional Review Board (IRB), Faculty of Medicine, Chulalongkorn University (IRB No. 0657/68).

## 3 Results

### 3.1 Sociodemographic Characteristics of Participants

Of the 400 participants, more than half were aged 18–30 years (55.8%), followed by those aged 31–45 years (39.5%); participants aged 46 years and above constituted a small proportion (4.8%). The mean age of the sample was 30.86 years (SD = 7.66). The sample was slightly male‐predominant (53.0%). Educational attainment was high, with most respondents reporting undergraduate (39.8%) or postgraduate (35.5%) education.

Nearly two‐thirds of participants were employed full‐time (64.0%), and marital status was evenly distributed between single (49.2%) and married individuals (48.5%). Most respondents reported a monthly income of ≤300,000 Nigerian Naira (67.0%), whereas smaller proportions reported no income (11.2%) or declined to disclose income (9.0%).

Geographically, participants were more frequently from Northern Nigeria (75.8%) than from the South (24.2%). Ethnic representation was diverse, with Hausa (35.8%), Yoruba (15.5%), Igbo (9.5%), and other ethnic groups (39.2%) represented. Religious affiliation was broadly balanced, with 52.8% identifying as Muslims and 47.2% as Christians (Table 1).

**TABLE 1:** Sociodemographic characteristics of participants (*N* = 400).

Variable	Frequency (%)
***N***	400
**Age group (years)**	
18–30	223 (55.8)
31–45	158 (39.5)
46 and above	19 (4.8)
Mean ± SD (years)	30.86 ± 7.66
**Gender**	
Female	188 (47.0)
Male	212 (53.0)
**Highest educational level**	
Primary	1 (0.2)
Secondary	17 (4.2)
Diploma	81 (20.2)
Undergraduate	159 (39.8)
Postgraduate	142 (35.5)
**Employment status**	
Unemployed	84 (21.0)
Full‐time	256 (64.0)
Part‐time/casual worker	58 (14.5)
Retired	2 (0.5)
**Marital status**	
Single (never married)	197 (49.2)
Married	194 (48.5)
Divorced	4 (1.0)
Widowed	5 (1.2)
**Monthly income (Nigerian Naira)**	
≤300,000	268 (67.0)
301,000–500,000	25 (6.2)
≥501,000	26 (6.5)
No income	45 (11.2)
Refused to answer	36 (9.0)
**Region**	
North	303 (75.8)
South	97 (24.2)
**Ethnicity**	
Hausa	143 (35.8)
Yoruba	62 (15.5)
Igbo	38 (9.5)
Other ethnicities	157 (39.2)
**Religion**	
Christianity	189 (47.2)
Islam	211 (52.8)

### 3.2 Comparison of Perception on HLM Across Religions and Age Group

Perception scores regarding HLM differed significantly by religious affiliation (Table 2). Christian respondents had a higher mean perception score (mean 4.39, SD 1.01; 95% confidence interval [CI] 4.25–4.54) compared with Muslim respondents (mean 4.04, SD 1.09; 95% CI 3.89–4.19), where Christianity shows higher mean ranks than Islam (Mann–Whitney *U* test: *z* = 3.882, *p* < 0.001). Additionally, the effect size was *r* = 0.194 (*z*/√*N*) and a Cohen's *d* of approximately 0.33.

**TABLE 2:** Perception scores for HLM by religion and age group (*N* = 400).

Domain	Group	*n*	Mean ± SD	95% CI (mean)	Rank sum	Mean rank	Test statistic	*p* value
Religion	Christianity	189	4.39 ± 1.01	4.25–4.54	41,969.00	221.00		
	Islam	211	4.04 ± 1.09	3.89–4.19	38,231.00	181.19	*z* = 3.882	<0.001^*^
Age group (years)	18–30	223	—	—	44,302.50	198.67		
	31–45	158	—	—	31,270.50	197.91	*χ* ^2^ = 3.347	0.188^**^
	≥46	19	—	—	4627.00	243.53		

*Note:* Perception score is the mean of the contributing 5‐point scale ranging from 1 to 5.

Abbreviation: CI, confidence interval; HLM, Healthy Longevity Medicine.

* *p* value from the two‐sided Mann–Whitney *U* test comparing religions.

** *p* value from the Kruskal–Wallis test comparing perception scores across age groups.

Perception scores for HLM did not differ significantly across age groups. Although participants aged ≥46 years had the highest mean rank, the overall comparison among the 18–30, 31–45, and ≥46 years groups was not statistically significant (Kruskal–Wallis test, *p* = 0.188). Given the small size of the ≥46‐year group, the higher mean rank should be interpreted with caution. Overall, perception levels were broadly comparable across age categories in this study.

### 3.3 Factors Associated With Interest in HLM

Education and monthly income emerged as the main correlates of interest in HLM services among the study participants (Table 3). Taking diploma holders as the reference group, participants with an undergraduate qualification had lower odds of reporting interest in both univariable and multivariable models (crude odds ratio [cOR] 0.39; 95% CI 0.15–0.99; *p* = 0.048; adjusted odds ratio [aOR] 0.36; 95% CI 0.14–0.93; *p* = 0.036). Those with secondary or postgraduate education did not differ significantly from the reference group, and estimates for primary education could not be computed because of sparse data.

**TABLE 3:** Univariable and multivariable logistic regression analysis of factors associated with interest in HLM services (*N* = 400).

	Univariable analysis		Multivariable analysis	
Variable	Crude OR (95% CI)	*p* value	Adjusted OR (95% CI)	*p* value
**Religion**				
Christian (Ref)	1.00	—	1.00	—
Islam	1.08 (0.60–1.95)	0.786	0.84 (0.41–1.73)	0.642
**Age group (years)**				
18–30 (Ref)	1.00	—	1.00	—
31–45	2.08 (1.06–4.07)	0.033	2.03 (0.94–4.36)	0.070
≥46	0.99 (0.27–3.59)	0.991	1.18 (0.29–4.78)	0.816
**Education level**				
Primary^a^	Not estimated	—	Not estimated	—
Secondary	0.60 (0.11–3.26)	0.554	0.74 (0.13–4.30)	0.739
Undergraduate	0.39 (0.15–0.99)	0.048	0.36 (0.14–0.93)	0.036
Postgraduate	0.63 (0.24–1.68)	0.356	0.57 (0.19–1.69)	0.309
Diploma (Ref)	1.00	—	1.00	—
**Monthly income (₦)**				
≤300,000 (Ref)	1.00	—	1.00	—
301,000–500,000	0.37 (0.14–1.00)	0.050	0.31 (0.11–0.90)	0.032
≥501,000	1.40 (0.31–6.24)	0.659	1.41 (0.30–6.51)	0.661
No income	0.76 (0.29–1.95)	0.566	0.94 (0.35–2.55)	0.909
Refused to answer	0.35 (0.15–0.82)	0.015	0.30 (0.12–0.76)	0.011
**Region**				
North (Ref)	1.00	—	1.00	—
South	0.59 (0.32–1.12)	0.108	0.55 (0.25–1.19)	0.130

*Note:* Odds ratios above 1.00 indicate greater interest relative to the reference category. Each variable was first modeled separately, then all five variables were entered simultaneously in the multivariable model.

Abbreviations: CI, confidence interval; HLM, Healthy Longevity Medicine; OR, odds ratio; Ref, reference category.

^a^ Primary education predicted the outcome perfectly, so its odds ratio could not be estimated and one observation was dropped. Univariable models were fitted on 400 observations, except education level (*N* = 399). The multivariable model was fitted on 399 observations. Model fit: Hosmer–Lemeshow *χ* ^2^ [7] = 6.80, *p* = 0.5580 (10 groups); area under the receiver operating characteristic curve (AUC) = 0.7121 (95% CI 0.64370–0.78046). Variance inflation factors: 1.06–1.52 (mean 1.22).

Compared with participants earning ₦300,000 or lower per month, those earning ₦301,000–₦500,000 had about one‐third the adjusted odds of reporting interest (aOR 0.31; 95% CI 0.11–0.90; *p* = 0.032), an association that was not significant before adjustment (cOR 0.37; 95% CI 0.14–1.00; *p* = 0.050). Participants who declined to state their income also had lower odds in both models (cOR 0.35; 95% CI 0.15–0.82; *p* = 0.015; aOR 0.30; 95% CI 0.12–0.76; *p* = 0.011). Earning ₦501,000 or higher and reporting no income were not associated with interest.

Participants aged 31–45 years had about twice the odds of interest in the crude model (cOR 2.08; 95% CI 1.06–4.07; *p* = 0.033), but this was no longer significant after adjustment for educational level, monthly income, and region (aOR 2.03; 95% CI 0.94–4.36; *p* = 0.070). Age 46 years and above, religious affiliation and region showed no significant associations in either model, indicating similar levels of expressed interest across the two religious groups despite differences in mean perception scores (Table 3). Additionally, the multivariable model showed no evidence of poor fit (Hosmer–Lemeshow *χ* ^2^(8) = 6.80, *p* = 0.5580) and acceptable discrimination (AUC = 0.7121, 95% CI 0.64370–0.78046; *n* = 399). VIFs ranged from 1.06 to 1.52 (mean 1.22), indicating no meaningful multicollinearity. The distribution of response intensity further supported this overall receptiveness: across both Christianity and Islam, responses clustered toward affirmative options rather than negative ones (Figure 2).

**FIGURE 2:**
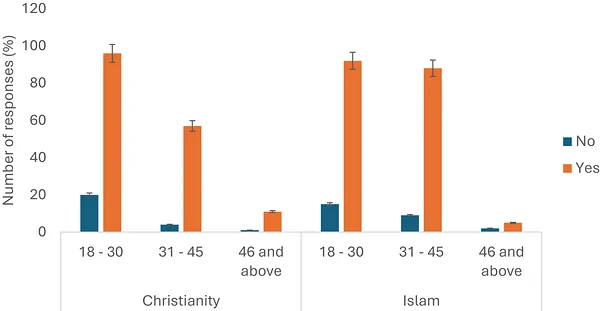
Distribution of participants' interest in HLM services by age group and religion (*N* = 400). Bars show the percentage of respondents within each religion and age subgroup who reported interest (Yes) and no interest (No) in using HLM services across three age groups (18–30, 31–45, and ≥46 years) among Christian and Muslim participants. Subgroup denominators are shown beneath each pair of bars. Error bars indicate ±1 standard error of the proportion.

## 4 Discussion

The study examined perceptions of HLM and interest in HLM services among Nigerian adults, with particular attention to differences by religious affiliation and age. Overall, respondents reported high levels of receptiveness toward HLM, indicating broad openness to longevity‐focused healthcare among this sample of Nigerian adults. Although perception scores differed significantly by religion, expressed interest in HLM services was high across both Christian and Muslim participants, and socioeconomic position, rather than religion or age, emerged as the main correlate of interest once other factors were considered. These findings are exploratory. The sample was not representative, the questionnaire has not been validated in Nigeria, and the perception score had modest internal consistency, so the patterns described here should be read as hypotheses for further study.

Christian respondents had higher perception scores than Muslim respondents, with higher mean ranks from the Mann–Whitney *U* test. This finding should be interpreted cautiously with regard to whether the observed difference arises from intrinsic doctrinal differences between Christianity and Islam. Literature from other settings has associated Christian traditions with language about stewardship of health and Islamic traditions with an emphasis on divine will and acceptance [19]. However, the evidence is drawn from populations whose health systems, histories, and religious institutions differ from those of Nigeria, so its transfer to this setting is tentative. In addition, the relationship between religion and health attitudes is highly context‐dependent and sensitive to how religious constructs are defined and operationalized in research [20]. Therefore, our findings do not serve as an evidence of doctrinal difference between the two faiths. The difference should also be read, considering the modest internal consistency of the perception score. It may reflect views on one of the three interventions rather than a general orientation toward HLM.

Perception mean scores did not differ significantly across age groups. This is consistent with the existing evidence rather than at odds with it. A cohort study of attitudes to life extension found no difference in willingness to use such treatment across young, younger old, and older old adults, and the oldest group was in fact the most likely to decline [21]. Reviews of public attitudes to longevity biotechnology likewise reported both interest in and ambivalence rather than enthusiasm that rises with age [22]. The intuition that older adults should be more receptive because their health needs are greater is therefore not well supported, and we do not treat our null result as surprising.

Midlife offers the more informative comparison. In our findings, participants aged 31–45 years had the highest odds of expressing interest before adjustment, although the association weakened once education and income were taken into account, whereas the youngest and oldest groups did not differ from one another. The Singapore HELO survey also found interest concentrated below the older end of the adult range, reporting that younger age was associated with stronger interest in HLM clinics [23]. The two findings are not identical. Singapore reported a broadly monotonic relationship, whereas ours peaks in midlife, and the two samples differ markedly in age structure. What the studies share is that interest does not rise with age.

Returning to perception, the absence of an age difference may also partly reflect the small size of the oldest age group (≥46 years), which limited statistical power. It may also reflect a tendency for adults across age groups to prioritize interventions with immediate relevance to current well‐being rather than long‐term health outcomes. Although participants aged ≥46 years had the highest mean rank for perception, this pattern should be interpreted cautiously and warrants further investigation in studies with larger and more age‐balanced samples.

Notably, despite differences in perception scores by religion, religious affiliation was not associated with interest in HLM services after adjustment for education, monthly income, and region. Both Christian and Muslim participants expressed similarly high levels of interest, suggesting that willingness to engage with longevity‐focused services may transcend religious differences when perceived benefits are salient. This distinction between perception and interest is important: Religious beliefs may be linked to how HLM is conceptualized, but not necessarily to whether individuals are willing to engage with such services. Similar patterns have been observed in other healthcare contexts, where service uptake is more closely linked to perceived personal benefit and health risk awareness than to religious affiliation alone [24]. However, this contrasts with findings from Singapore, where Christianity was associated with greater interest in attending HLM clinics [23], underscoring the importance of sociocultural context.

In the regression analysis, education and monthly income were the variables most clearly associated with interest in HLM services after adjustment. The attenuation suggests that midlife interest may be bound up with schooling and earnings rather than with age or religion. Participants holding an undergraduate qualification had lower odds of reporting interest than diploma holders in our study, and a similar pattern appeared among those earning ₦301,000–₦500,000 each month. This runs counter to the Singapore HELO survey, where higher education and higher income were linked to stronger interest in HLM clinics [23]. Cost may account for part of the difference, because patients surveyed at a private hospital in Abuja named cost as the leading barrier to preventive care, ahead of distance and lack of insurance [25]. However, this remains speculative in this study because willingness‐to‐pay items were not analyzed and reported. Hence, these data cannot show whether cost shaped the responses observed. Additionally, the diploma reference group often represents a distinct educational and socioeconomic category in Nigeria. Diploma holders from polytechnics and other professional institutions may differ from university graduates in healthcare experiences, occupations, and use of preventive services. These unmeasured factors may underlie the differences observed. Thus, the findings should not be taken to mean that diploma education reduces interest in HLM. However, they underscore the need for research on how educational pathways, socioeconomic position, healthcare access, and health beliefs interact to shape interest in longevity‐oriented services in Nigeria. Finally, the significantly lower odds of interest among respondents who declined to state their income should be viewed with caution. Refusal on income items tends to track respondent characteristics rather than occur at random; this group is therefore best treated as a mixed category rather than as an income band.

The Singapore comparison drawn on at several points above should be treated as indicative rather than direct. The two settings differ in nearly every dimension relevant to how longevity medicine is encountered (Table 4). Singapore is a high‐income, entirely urban city‐state with universal health coverage and a mature private health sector, whereas Nigeria is a lower middle‐income country, roughly half rural, in which health financing depends heavily on out‐of‐pocket expenditure. The samples differ as well, with a median age of 46 years in Singapore against a mean of 31 years here, random door‐to‐door and stratified online sampling, and with a probability‐based design there against convenience recruitment for our case. Interest in HLM also differed, at 55.5% in Singapore and 87.3% here. A self‐selected online sample is likely to overstate interest, so part of this gap may reflect sampling rather than setting. Where the two studies agree, as on interest not rising with age, the agreement is worth noting. Where they diverge, as on the association between Christianity and interest, the divergence cannot be attributed to religion alone, because the health systems, age structures, and sampling designs all differ at the same time.

**TABLE 4:** Comparison of the settings and designs of the Singapore HELO survey and the present study.

Characteristic	Singapore HELO survey [22]	Present study (Nigeria)	Source
**Setting**			
World Bank income group	High income	Lower middle income	https://datahelpdesk.worldbank.org/knowledgebase/articles/906519
GDP per capita, current US$ (2024)	90,674	1,084	https://data.worldbank.org/indicator/NY.GDP.PCAP.CD?locations=SG‐NG
Life expectancy at birth, years (2023)	82.9	54.5	https://data.worldbank.org/indicator/SP.DYN.LE00.IN?locations=SG‐NG
Population aged 65 years and above, % (2024)	13.7	3.0	https://data.worldbank.org/indicator/SP.POP.65UP.TO.ZS?locations=SG‐NG
Urban population, % (2024)	100	55.0	https://data.worldbank.org/indicator/SP.URB.TOTL.IN.ZS?locations=SG‐NG
Out‐of‐pocket share of current health expenditure, % (2023)	25.4	70.9	Singapore: https://www.commonwealthfund.org/international‐health‐policy‐center/countries/singapore Nigeria: https://www.commonwealthfund.org/international‐health‐policy‐center/countries/nigeria
Health insurance coverage	Universal; MediShield Life covers all citizens and permanent residents	Fewer than 10% of citizens insured (2024)	Singapore: https://www.commonwealthfund.org/international‐health‐policy‐center/countries/singapore Nigeria: https://www.commonwealthfund.org/international‐health‐policy‐center/countries/nigeria
**Study design**			Singapore: https://doi.org/10.1007/s11357‐026‐02160‐8 Nigeria: present study
Data collection	June to August 2024	November to December 2025	
Sampling	Multistage cluster sampling of postal codes, door‐to‐door (*n* = 2003), and a stratified online panel (*n* = 1031)	Online convenience and snowball sampling through social media	
Participants analyzed	3034	400	
Minimum age	21 years	18 years	
Survey languages	English, Chinese, Malay, and Tamil	English only	
Incentive	S$20 door‐to‐door; panel rewards online	None	
Data quality exclusions	289 failing a completion threshold	29,996 likely automated submissions at Stages 1 and 12 at Stage 2	
Analysis of interest	Crude and age‐adjusted logistic regression	Crude and multivariable logistic regression, with religion, age, education, income, and region entered together	
**Sample and findings**			Singapore: https://doi.org/10.1007/s11357‐026‐02160‐8 Nigeria: present study
Age	Median 46 years (IQR 34–59)	Mean 30.86 years (SD 7.66)	
Tertiary education	31.1% undergraduate; 14.6% postgraduate	39.8% undergraduate; 35.5% postgraduate	
Interest in HLM	55.5% interested in attending an HLM clinic	87.3% (349/400) interested in HLM services	
Factors associated with interest	Younger age, male gender, Christianity, higher education, full‐time employment, and higher household income associated with greater interest	Religion, age, and region are not associated after adjustment; undergraduate education, a monthly income of ₦301,000–₦500,000, and declining reported income are associated with lower odds	

*Note:* World Bank indicators are from the World Development Indicators. Out‐of‐pocket shares in the Commonwealth Fund profiles are drawn from the WHO Global Health Expenditure Database (https://apps.who.int/nha/database).

Abbreviations: GDP, gross domestic product; HELO, Healthy Longevity; HLM, Healthy Longevity Medicine; IQR, interquartile range; SD, standard deviation; S$, Singapore dollars.

## 5 Study Limitations

The most important limitation of this study is its sampling design. Participants were recruited by snowball sampling through social media, which is a non‐probability method, limiting wider national representativeness. Recruitment began from seed participants and our own social media accounts, so respondents are likely to resemble those networks, and a response rate cannot be calculated for a snowball sample. The sample departs from the national profile on several characteristics at once. It is young, with 55.8% aged 18–30 years and only 4.8% aged 46 or above. It is highly educated, with 75.3% holding undergraduate or postgraduate qualifications. It is heavily Northern, at 75.8%. Its reported income is concentrated in a single wide band above Nigeria's national minimum wage of ₦70,000, with 67.0% selecting ₦300,000 or less per month. Participation further required English literacy, internet access, and presence on the recruitment platforms, which placed roughly half of Nigerian adults outside the reach of the survey by design. These departures all run in the same direction, toward younger, better connected, more affluent, and formally employed Nigerians, so they are likely to compound rather than offset one another. In addition, the monthly income variable is best read as a marker of that non‐representativeness, and the income associations reported in Table 3 should be interpreted within this narrow and unevenly distributed range. Because education, income, and internet access are each plausibly related to attitudes toward preventive and longevity‐oriented healthcare, the high overall interest observed here probably overstates interest in the wider population. Religion and region are also closely aligned in Nigeria, so the religious difference in perception scores may partly reflect regional history and access to health services rather than belief alone. The group aged 46 years and above was too small to support inference. Even if every respondent in that group had expressed interest, statistical power would have remained low. The same constraint applies to the perception analysis, where the oldest group recorded the highest mean rank. Additionally, the cross‐sectional design precludes causal inference, and the study did not examine differences across denominations within Christianity or Islam. Future research should address these limitations through more representative sampling, longitudinal designs, and finer grained analyses of religious heterogeneity.

The HELO questionnaire, originally developed and validated in high‐income settings such as Singapore, was chosen for this study because its core constructs (lifespan, healthspan, awareness, perceptions, and interest in HLM) are pertinent to population aging and chronic disease prevention in Nigeria. However, we acknowledge that an instrument developed largely outside Nigeria and where its validity in the Nigerian context has not been formally tested may not fully capture all sociocultural meanings of longevity, aging, and healthcare preferences in the Nigerian context. Moreover, because the survey was administered exclusively in English, language barriers may have prevented the participation of people who hold different views on HLM. This linguistic and accessibility limitation may affect the generalizability of the findings, as responses may reflect the perceptions and interests of more educated, high‐income, and digitally connected Nigerians rather than the entire adult population.

A further limitation is the reliability of the perception score. It comprised three items, and Cronbach's alpha was 0.60, which is below the conventional threshold of 0.70 for internal consistency. The mean inter‐item correlation was 0.31, and item‐rest correlations ranged from 0.34 to 0.44. Two points qualify this value. Alpha rises with the number of items, so a lower value is expected for a three‐item measure. The three items also asked about conceptually distinct interventions (increasing lifespan alone, increasing healthspan alone, and increasing both), and respondents may reasonably hold different views about each, so strong agreement between items is not necessarily expected. Even so, the value indicates that the items may not measure a single underlying construct reliably. This has three implications. First, random measurement error in the score would reduce the precision of the comparisons by religion and age group. Second, the composite may combine different attitudes, so the higher mean score among Christian respondents may reflect a difference on one of the three interventions rather than a general orientation toward HLM. In addition, no factor analysis or other psychometric validation was carried out in this sample, and the HELO items have not been formally validated in Nigeria. Findings involving the perception score should therefore be treated as provisional, and the score should not be regarded as a validated measure of perceptions of HLM in Nigeria. The interest outcome was a single yes/no item, so its reliability could not be assessed. Contextual adaptation and psychometric validation are recommended before the scale is used again in Nigeria.

Moreover, the willingness‐to‐pay variables included in the survey questionnaire were not analyzed in the present study, as they were beyond the primary objectives of this investigation. Therefore, no measure of affordability, ability to pay, or price sensitivity underpins any result reported here, and this study cannot show whether cost shapes interest in HLM services. Future studies should consider analyzing willingness to pay in greater detail to better understand affordability considerations.

The screening procedure is a major limitation of this study. Because IP addresses and response times were not available, classification of a submission as automated rested on indicators, including attention check questions, an empty response record, and timestamps. IP addresses and response times are regarded as the strongest indicators of automated responding. The screening was reasonable given the data that Google Forms provides, but it did not meet that standard. It is therefore possible that some of the 412 responses that passed Stage 1, and so some of the 400 analyzed, were also automated or were duplicate submissions. A bot could have completed the questionnaire instead of submitting it empty, and one person could have submitted more than once using different email addresses. Neither could be detected with the data available. The findings should therefore be treated as exploratory. Future online surveys in this regard should use a platform that records IP address and response duration.

## 6 Recommendations

Future studies should recruit larger, more age‐balanced, and regionally representative samples and consider longitudinal designs to assess how perceptions and interest in HLM evolve across the life course. Because education and income, rather than religion or age, were most clearly associated with interest in this study, socioeconomic position deserves to be treated as a primary exposure in the future. Willingness‐to‐pay items were collected in this survey but not analyzed, and analyzing them, alongside direct measurement of ability to pay, is an obvious next step. This is needed because cost was the leading barrier named by patients surveyed about preventive care in Abuja [25] and catastrophic health spending in Nigeria falls hardest on households of lower socioeconomic status and on those without insurance [26]. Until it is done, the role of affordability in shaping interest in HLM remains untested. Research should also explore denominational and doctrinal diversity within Christianity and Islam to better understand intra‐religious variation in health perceptions. Beyond the research agenda, several practical steps follow from these findings. Nigeria already has substantial faith‐based health infrastructure that could carry longevity‐oriented messaging without building new channels.

Framing should be adapted to each tradition rather than standardized across them. For Christian audiences, HELO aligns readily with existing language about stewardship of the body and about health as a blessing to be actively pursued. For Muslim audiences, framing matters more. Presenting HLM as the prevention and treatment of disease, and as preserving the capacity to fulfill religious and family obligations in later life, sits within established Islamic teaching on seeking treatment. Engaging imams and church leaders as intermediaries would be prudent in both cases.

Targeting should follow socioeconomic position as much as age. The midlife pattern held only before adjustment, so on its own it is too weak to justify concentrating services on adults aged 31–45 years. What the models show more clearly is that interest varied with education and income. Whether this variation reflects affordability cannot be determined from these findings, because willingness to pay was not analyzed. Cost and the absence of insurance are recurrent constraints on healthcare in Nigeria, so financial protection is worth investigating. Widening financial protection may therefore matter more than age‐based targeting, given how consistently cost and the absence of insurance appear as constraints in Nigeria.

From a policy and practice perspective, the findings highlight the importance of culturally and religiously sensitive approaches to promoting HLM in Nigeria. Public health communication strategies should emphasize the compatibility of HLM with broadly shared values related to well‐being and functional independence, rather than framing longevity medicine in ways that may conflict with religious interpretations. Because interest did not differ by religion and, after adjustment, did not differ by age, there is little basis for restricting healthspan‐oriented services to any single demographic group. Priority is better placed on making such services widely available across groups.

## 7 Conclusion

This study indicates that while religious affiliation is associated with differences in perceptions of HLM among the study participants, it was not significantly associated with expressed interest in HLM services. After adjustment, education and monthly income were the variables significantly associated with interest, whereas the higher odds of interest seen among adults aged 31–45 years before adjustment did not persist. Undergraduate education, a monthly income of ₦301,000–₦500,000 and declining to report income were each associated with lower odds of interest. These findings underscore the need to distinguish between perception and service interest when designing and promoting longevity‐focused healthcare. They also suggest that education and income may shape engagement with HLM more than religion or age group, and implications for cost remain speculative. Public health strategies should pair culturally sensitive communication with further study of the socioeconomic factors that shape access across Nigeria's diverse population. Additionally, future research should use probability‐based sampling with enough older adults to support inference, analyze the willingness‐to‐pay data to assess affordability barriers, and validate the HELO questionnaire in Nigeria before its perception items are used.

## Author Contributions

**Shuaibu Saidu Musa**: investigation, data curation, data analysis, writing – original draft, writing – review and editing. **Mohamed Mustaf Ahmed**: investigation, data analysis, writing – review and editing. **Bukola Lawal**: writing – review and editing. **Angella Maliakini Katenga**: writing – review and editing. **Krit Pongpirul**: conceptualization, supervision, project administration, writing – review and editing. All authors have read and approved the final version of the manuscript.

## Funding

The authors have nothing to report.

## Conflicts of Interest

Krit Pongpirul is an editorial board member of Public Health Challenges and co‐author of this article. He is excluded from editorial decision‐making related to the acceptance of this article for publication in the journal.

## Data Availability

The datasets generated and/or analyzed during the current study are available from the corresponding author on reasonable request.
